# Geographic Pattern of Typhoid Fever in India: A Model-Based Estimate of Cohort and Surveillance Data

**DOI:** 10.1093/infdis/jiab187

**Published:** 2021-11-23

**Authors:** Yanjia Cao, Arun S Karthikeyan, Karthikeyan Ramanujam, Reshma Raju, Swathi Krishna, Dilesh Kumar, Theresa Ryckman, Venkata Raghava Mohan, Gagandeep Kang, Jacob John, Jason R Andrews, Nathan C Lo

**Affiliations:** 1 Division of Infectious Diseases and Geographic Medicine, Stanford University School of Medicine, Stanford, California, USA; 2 Wellcome Research Unit, Christian Medical College, Vellore, India; 3 Department of Community Health, Christian Medical College, Vellore, India; 4 Center for Health Policy and the Center for Primary Care and Outcomes Research, Stanford University School of Medicine, Stanford, California, USA; 5 Deparment of Medicine, University of California, San Francisco, San Francisco, California, USA

**Keywords:** enteric fever, geospatial model India, salmonella, typhoid fever, public health, vaccination

## Abstract

**Background:**

Typhoid fever remains a major public health problem in India. Recently, the Surveillance for Enteric Fever in India program completed a multisite surveillance study. However, data on subnational variation in typhoid fever are needed to guide the introduction of the new typhoid conjugate vaccine in India.

**Methods:**

We applied a geospatial statistical model to estimate typhoid fever incidence across India, using data from 4 cohort studies and 6 hybrid surveillance sites from October 2017 to March 2020. We collected geocoded data from the Demographic and Health Survey in India as predictors of typhoid fever incidence. We used a log linear regression model to predict a primary outcome of typhoid incidence.

**Results:**

We estimated a national incidence of typhoid fever in India of 360 cases (95% confidence interval [CI], 297–494) per 100 000 person-years, with an annual estimate of 4.5 million cases (95% CI, 3.7–6.1 million) and 8930 deaths (95% CI, 7360–12 260), assuming a 0.2% case-fatality rate. We found substantial geographic variation of typhoid incidence across the country, with higher incidence in southwestern states and urban centers in the north.

**Conclusions:**

There is a large burden of typhoid fever in India with substantial heterogeneity across the country, with higher burden in urban centers.

Typhoid fever has an estimated global incidence of 11–21 million cases annually, resulting in 120 000–160 000 deaths [1–4]. Enteric fever is an acute febrile illness caused by ingestion of the bacterium *Salmonella enterica* serotype Typhi (*S* Typhi) or serotype Paratyphi A, B or C, often through food or water contaminated with human feces [5, 6]. The severe clinical presentations of typhoid fever includes the development of sepsis, gastrointestinal bleeding, intestinal perforation, and death [7, 8].

A large proportion of the global burden of typhoid fever is concentrated in South Asia, with a high incidence in India [2–4, 9–14]. The Global Burden of Disease Study in 2017 estimated typhoid/paratyphoid incidence in India of 586 cases per 100 000 person-years [4, 15]; however, these estimates extrapolated largely from regional data, because there have been few population-based studies in India. A 1996 study in Delhi found an incidence of 976 (95% confidence interval [CI], 763–1250) cases per 100 000 person-years, whereas a 2006 study in Kolkata estimated an incidence of 265 (95% CI, 217–324) cases per 100 000 person-years [16, 17]. There have been no population-based typhoid incidence studies for more than a decade, and there have been no prior population-based studies from rural areas, where the majority of people reside. A recent meta-analysis, largely of facility-based studies, revealed that the proportion of individuals with positive blood cultures for *S* Typhi has been declining [10]. However, there remains scarce recent data on the incidence and geographic distribution of typhoid fever in India. This paucity of data is further complicated because the geographic pattern of typhoid fever is expected to be highly heterogenous within the country [10, 18, 19].

Accurate and recent estimates of typhoid fever incidence and the spatial distribution in India are essential for public health decision making such as vaccination strategies. The World Health Organization (WHO) recently approved new Vi conjugate vaccines against typhoid fever that provide high efficacy and duration of protection [20]. To address the need for locally relevant data for typhoid fever burden in India to guide policy on use of the conjugate vaccines in India, the Surveillance for Enteric Fever in India (SEFI) study was conducted [21, 22]. The SEFI is a multisite study that used both prospective cohorts and hybrid surveillance designs in 10 urban and rural locations to provide estimates on typhoid fever incidence. Although the SEFI study sites provide high-quality typhoid fever incidence data for these sites, there is a need for broader estimates of typhoid burden across India. The use of spatial modeling approaches has become increasingly common to predict epidemiologic measures (eg, incidence and prevalence) in infectious diseases (eg, malaria and schistosomiasis) in the absence of primary data [23–26]. This modeling approach aims to leverage variables from secondary datasets to predict incidence in areas without primary data on incidence, by calibrating the relationship of these variables with incidence in areas with primary data on incidence.

Although typhoid fever is common in India, the exact burden of disease and spatial heterogeneity are important to understand to guide policy decision on national vaccination with the Vi typhoid conjugate vaccines. To address this need, we applied a geospatial statistical model to estimate typhoid fever incidence across India, using data from 4 cohort studies and 6 hybrid surveillance from the SEFI study combined with a national household survey data.

## METHODS

### Methods Overview

We used a statistical model to predict typhoid fever incidence across India. We performed spatial data processing and interpolation to match health and demographic variables geographically to observed data on typhoid incidence from SEFI study sites. The model calibration and prediction both utilized regression analysis to estimate typhoid incidence, which was reported at a state and national level.

### Study Data on Typhoid Incidence

We used data on typhoid fever incidence from the SEFI study, which was a multisite cohort (named Tier 1) and hybrid surveillance (named Tier 2) study of typhoid incidence. The SEFI study had 10 sites that each provided a site-specific typhoid incidence estimate [21, 22]. In all study sites, typhoid cases were defined as blood culture-confirmed *S* Typhi cases over the duration of the study. The Widal test was not used for diagnosis. The spatial data for catchment areas at a village level for the 10 study sites in India were provided by the SEFI study. We used spatial information on each study site using ArcGIS 10.7.1.

The Tier 1 SEFI sites (cohort study) measured clinical typhoid fever cases and included 4 cohorts. Each cohort enrolled 6000 children ages 6 months to 15 years that were observed over a 2-year observation period (October 2017–February 2019). The study sites were located in Delhi, Kolkata (West Bengal), Vadu (Maharashtra), and Vellore (Tamil Nadu). The incidence of typhoid fever was computed as the number of blood culture-confirmed cases for each site, with the denominator being the number of person-years of observation in the defined age group, with follow up censored for 15th birthday, withdrawal of consent/assent, febrile period, death, and completion of study. The incidence estimate was reported as number of cases per 100 000 person-years. We adjusted our estimate to account for consent/assent to obtain blood cultures and blood culture sensitivity [21]. Further explanation of study methodology for Tier 1 has been previously described [27].

The Tier 2 SEFI sites (hybrid surveillance study) measured hospitalized typhoid fever cases, and included 6 hybrid surveillance sites. Each site measured the number of typhoid fever cases identified in the hospital in persons 6 months and older (including adolescents and adults) over a 2-year observation period (between February 2018 and March 2020). In each hybrid surveillance study site, healthcare utilization surveys were conducted to estimate the person-years of observation to adjust the catchment population denominator when computing incidence, with the methods as previously described [22, 28]. The incidence estimate was adjusted for the 60% sensitivity of blood cultures. The Tier 2 study sites were located in Chandigarh, Nandurbar (Maharashtra), Kullu (Himachal Pradesh), Karimganj (Assam), Anantapur (Andhra Pradesh), and East Champaran (Bihar). Among the 10 study sites, Delhi, Kolkata and Vellore in Tier 1 and Chandigarh in Tier 2 are urban areas, whereas the rest are rural locations. Details of the computation process for the incidence in these 10 study sites for Tier 1 and 2 are further described in the Appendix.

### Study Data on Model Predictors

The data on model predictors for typhoid fever incidence were drawn from the Demographic and Health Survey (DHS) conducted from 2015 to 2016 in India [29]. The DHS are nationally representative cross-sectional surveys on health and demographic variables that occur in many low- and middle-income countries approximately every 5 years [30]. The DHS have also been widely used by researchers and policymakers [31–33]. We extracted the following prespecified variables from the DHS that were chosen based on their potential to predict incidence of typhoid fever from prior literature: urbanicity (urban vs rural in a cluster, defined by the Indian national government), household wealth (quintile), household maternal education, household access to improved water and toilet, household size, household receipt of a third dose of the diphtheria-pertussis-tetanus vaccination (a marker of healthcare access), and anthropometric measurements (stunting and underweight). We simplified the definition of access to improved water and toilet following the Joint Monitoring Program for Water Supply, Sanitation and Hygiene [34] guidance. We defined stunting and being underweight based on the WHO Child Growth Standards using height-for-age and weight-for-age more than 2 standard deviations below a reference median, respectively. Population data were obtained from WorldPop [35]. Missing data were excluded. The variables were recoded and computed hierarchically as described in Supplementary Tables S1–S3 in the Appendix.

We performed a spatial interpolation of each DHS variable over India. Study variables were available at different levels including for each child, household, and cluster (see Supplementary Tables S2 and S3) and were each aggregated to a mean at the cluster level (ie, the primary sampling unit where the preexisting geographic area is known as census enumeration areas). For each cluster in the DHS survey, the GPS location (ie, points with latitude and longitude) at the center of each sample cluster was collected during field work or survey with variation for confidentiality inside the targeted administrative units (by up to 2 kilometers for urban locations and 10 kilometers for rural locations) [36]. The DHS survey for India in 2015 had a total of 28 395 clusters. We performed spatial interpolation using inverse distance weighting methodology for all variables from DHS (ie, cluster points) at 5 × 5-km resolution. The spatial resolution of 5 km was (1) chosen to line up with the smallest size of catchment area among the 10 study sites and (2) based on resolution of available datasets. The interpolation process was weighted by the number of households (or the number of children for certain variables) in each cluster. The spatial weights applied inverse distance with the power of 2 for the exponent of distance that controlled the significance of surrounding points on the interpolated value (ie, the weight of known points on unknown locations diminished with distance). The interpolation results were evaluated through cross-validation. The cross-validation process separated the data into a training set to calibrate the model and a test set to evaluate the predictive performance on data that were withheld from the model during the calibration. The raster output from inverse distance weighting methodology was then converted to 5 × 5-km polygon vector data to spatially intersect with study sites. The spatial data processing was implemented in ArcGIS 10.7.1.

### Model Calibration

We utilized a log linear regression model to estimate the relationship between typhoid incidence (dependent variable, cases per 100 000 person-years) and predictors (independent variables). We estimated the regression on the level of the study site, including the sample size of the 10 SEFI sites. For each predictor, the variable population mean of the DHS clusters overlying each SEFI study site was estimated. We selected variables by identifying the lowest Akaike Information Criterion (AIC) value, with a goal of limiting to a single variable to prevent overfitting given the limited sample size; in sensitivity analysis, we evaluated a 2–3 variable model. The final model was calibrated to the selected variable(s) at the level of the 10 SEFI sites.

### Model Prediction

**Figure 1.**
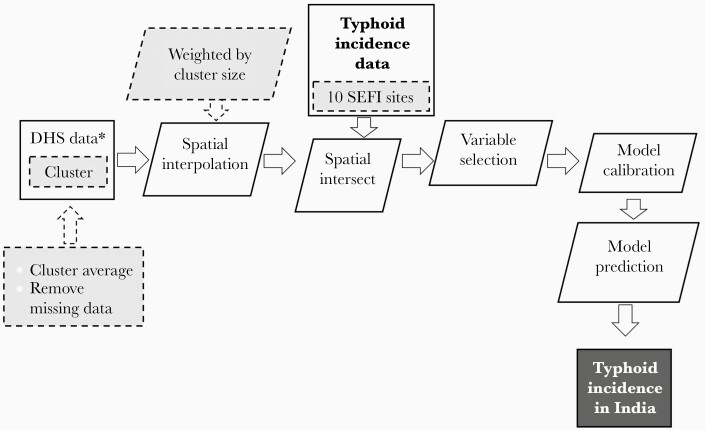
Summary of the study design for prediction of typhoid incidence in India. The study design followed the outlined process in the figure. We used Demographic and Health Survey (DHS) data on model variables to serve as predictors of typhoid incidence. The DHS variable data were averaged at a cluster level and then interpolated on a 5 × 5-km grid. We geographically intersected the DHS model variable data with the Surveillance for Enteric Fever in India (SEFI) data on observed typhoid incidence. We calibrated a model to estimate the relationship between each DHS model variable and typhoid incidence, and then we utilized a backward selection algorithm for variable selection. When the Akaike Information Criterion was minimized, we used the selected variable(s) as the predictor of typhoid incidence for the model. The rectangles refer to input/output data. The rhomboid shape refers to data processing. The gray shaded color indicates that additional data/processing steps.

We utilized the calibrated log linear regression to predict the primary study outcome of typhoid incidence (cases per 100 000 person-years) at the level of a 5 × 5-km grid using the selected variable. There was a total of 160 800 grids in India. The typhoid incidence estimate was aggregated to a state level, with population weighting for each grid. Statistical analyses were implemented in R 3.6.1. The methodology of this study is presented in Figure 1.

We computed the 95% uncertainty interval (UI) for the prediction of typhoid fever incidence using a resampling process that accounted for the uncertainty in the original SEFI site estimates of typhoid incidence. The process first sampled from the 95% interval on typhoid incidence for each SEFI site, which was bounded by a beta distribution. We then recalibrated the model using the 10 SEFI study site incidence estimates, repeated the typhoid incidence prediction for India, and stored the mean estimate. This process was repeated 1000 times to ensure convergence of the estimate. The final 95% UI in this study was based on the 2.5% and 97.5% percentile of this range of values. We also computed secondary study outcomes including (1) total number of national annual typhoid fever cases based on a population weighted incidence and (2) annual mortality based on a 0.2% case-fatality rate [12].

To test robustness in this analysis, we performed multiple sensitivity analyses. These included alternative variable selections and a leave-one-out analysis to determine the effect of removing 1 study site on the overall typhoid incidence prediction.

### Ethic Statement

This project did not meet the definition of human subjects research at Stanford University given use of aggregated estimates of typhoid fever incidence without identifiable or person-level data. In the SEFI study, all participants provided informed consent with institutional review board approval at Christian Medical College, Vellore, as well as approval at each study site.

## RESULTS

### Surveillance for Enteric Fever in India Data on Typhoid Incidence

**Figure 2.**
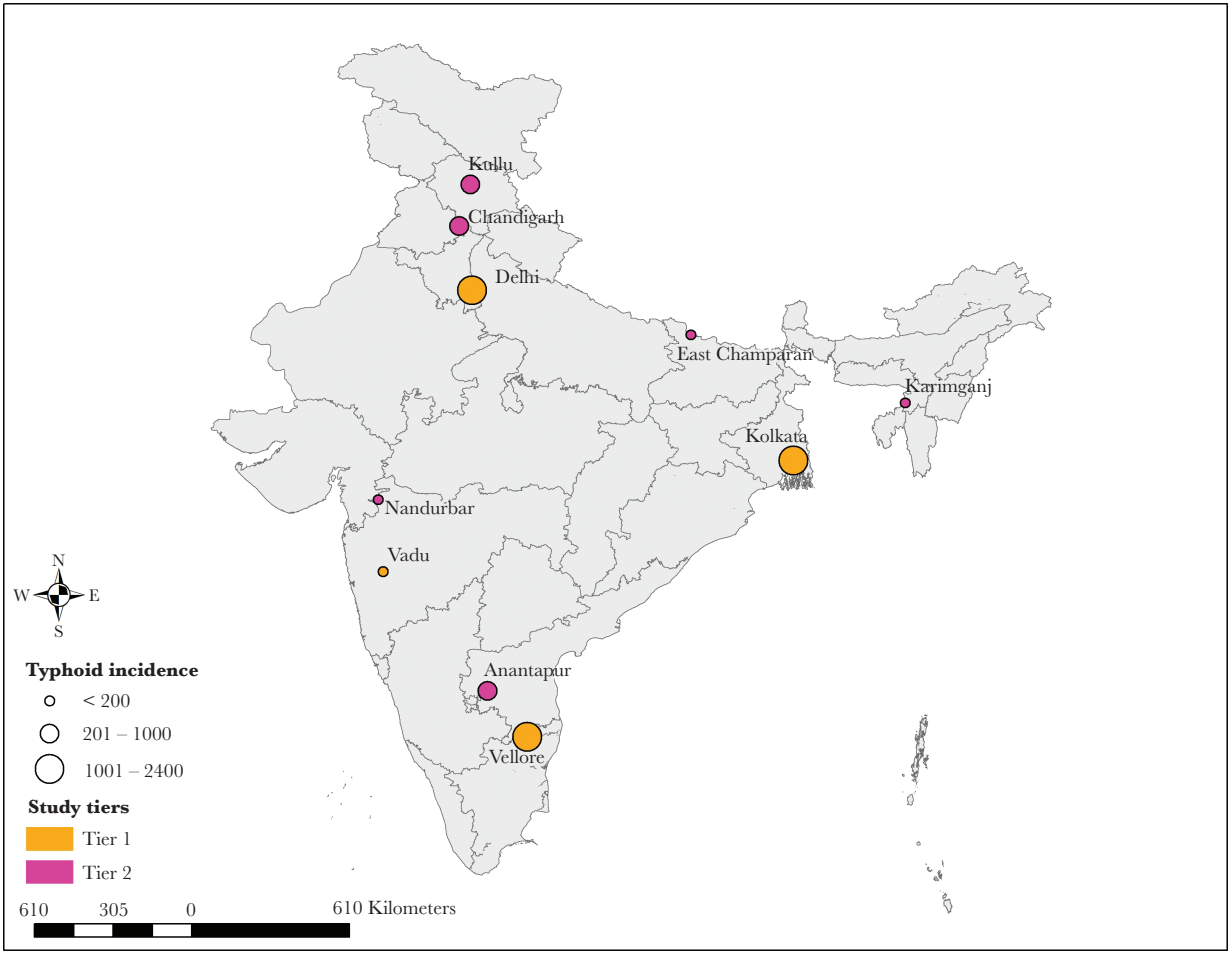
Spatial distribution of the 10 Surveillance for Enteric Fever in India (SEFI) study sites in India. The circles indicate the location of the 10 SEFI sites. The pink circles refer to 4 cohort study sites in Tier 1, and orange circles refer to 6 hybrid surveillance surveillance study sites in Tier 2. The size of the circles were categorized in 3 levels of incidence: fewer than 200 cases per 100 000 person-years (small circle), 201–1000 cases per 100 000 person-years (medium circle), and over 1000 cases per 100 000 person-years (large circle).

The overall mean incidence of typhoid fever in the 10 SEFI sites was 615 cases per 100 000 person-years. The location of the 10 sites and corresponding typhoid incidence is displayed in Figure 2. Typhoid incidence was higher in the cohort studies (Tier 1) with an average of 1080 cases per 100 000 person-years compared with the hybrid surveillance study (Tier 2) with an average of 304 cases per 100 000 person-years. The study sites in urban settings had a higher mean incidence of typhoid fever of 1310 cases per 100 000 person-years, compared with rural sites, which had a mean incidence of 151 cases per 100 000 person-years. Among the 10 sites, 3 had typhoid incidence below 100 cases per 100 000 person-years: 61 (95% UI, 24–125) in Vadu, 72 (95% UI, 50–133) in East Champaran, and 79 (95% UI, 59–133) in Karimganj. In addition, 3 sites had an incidence between 100 and 500 cases per 100 000 person-years: 154 (95% UI, 98–280) in Nandurbar, 266 (95% UI, 176–412) in Anantapur, and 274 (95% UI, 179–443) in Kullu. Finally, 4 sites had an incidence of over 500 cases per 100 000 person-years: 981 (95% UI, 717–1416) in Chandigarh, 1095 (95% UI, 913–1302) in Delhi, 1187 (95% UI, 998–1400) in Kolkata, and 1977 (95% UI, 1740–2236) in Vellore.

### Variable Selection and Model Calibration

The spatial interpolation of each DHS variable is available in Supplementary Figure S1 in the Appendix. The prediction error statistic (root mean squared error) from the inverse distance weighting interpolation for each DHS variable was also displayed in Supplementary Figure S1. We performed a variable selection process of DHS variables to predict typhoid fever incidence, and identified that urban prevalence minimized the AIC. The result from the model calibration using DHS variables is summarized in Table 1. The calibrated model was able to broadly reproduce the estimated pattern of typhoid incidence in many observed settings (Table 3).

**Table 1.** Univariate Regression on Predictors of Typhoid Fever Incidence

	Univariate Regression		
Variables	Coefficient	95% CI	AIC
Urban prevalence_a_	2.83	(2.47–3.18)	4.24
Improved toilet access (binary)	2.72	(0.15–5.29)	34.35
Education level (tertile)	1.45	(−0.76 to 3.55)	36.77
Access to vaccination (3rd dose, diphtheria, tetanus, and pertussis)	6.54	(1.98–11.11)	31.79
Wealth (quintile)	0.78	(0.19–1.37)	32.61
Household size	0.02	(−0.96 to 1.00)	38.64
Improved water access (binary)	0.62	(−4.99 to 6.24)	38.59
Stunting prevalence	−7.68	(−11.96 to −3.39)	29.31
Underweight prevalence	−3.36	(−8.15 to 1.41)	36.51

Abbreviations: AIC, Akaike Information Criterion; CI, confidence interval.

The model in the univariate regression was a log linear regression using the 10 study sites (N = 10). In this regression test, the dependent variable was typhoid incidence at each study site (cases per 100 000 person-years). The coefficient represents a log transformation.

^a^Urban prevalence was computed as the average of a binary urban/rural household variable at the cluster level.

### Model Prediction of Typhoid Incidence

**Figure 3.**
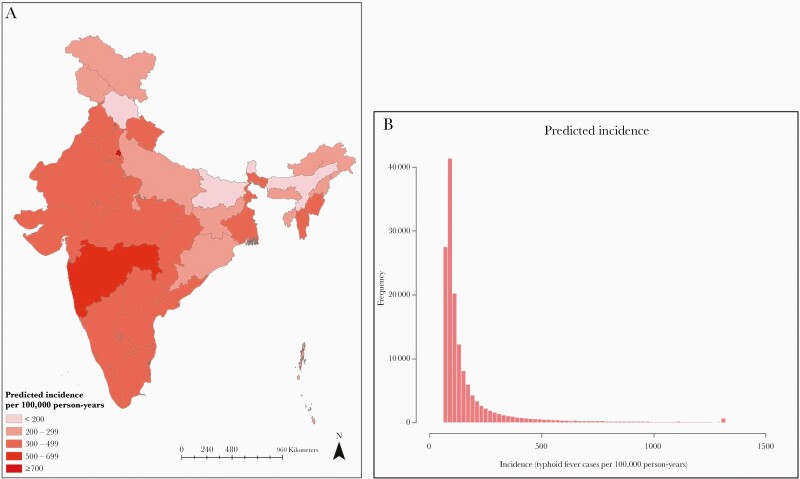
Predicted incidence of typhoid fever in India. We calibrated a statistical model to predict typhoid fever incidence in India using data from 10 Surveillance for Enteric Fever in India study sites. We used a log linear regression model to predict typhoid incidence across the country using secondary data obtained from Demographic and Health Survey in India. The estimated incidence was at 5 × 5-km grid level and was aggregated at state level and mapped in (a). The histogram of incidence at original grid level was visualized in (b).

The national incidence of typhoid fever was estimated to be 360 cases per 100 000 person-years (95% UI, 297–494), adjusted for blood culture sensitivity. Based on this incidence, we estimated 4.5 million (95% UI, 3.7–6.1 million) annual cases of typhoid fever with approximately 8930 deaths (95% UI, 7360–12 260), assuming a 0.2% case-fatality rate. The mean typhoid incidence in urban settings was 770 cases per 100 000 person-years (95% UI, 620–1040), whereas the mean incidence was 150 cases per 100 000 person-years (95% UI, 130–210) in rural settings. We noted there was substantial variation in predicted typhoid incidence across the country in Figure 3. The incidence ranged from 149 cases per 100 000 person-years (95% UI, 130–213) for Himachal Pradesh to 1245 cases per 100 000 person-years (95% UI, 963–1702) for Delhi. Table 2 summarized the predicted incidence for each state as well as the prevalence of state urbanicity. In general, there was higher incidence in southern and western states (eg, Maharashtra and Tamil Nadu) and urban centers in the north (eg, Delhi and Chandigarh), whereas there was lower incidence in rural northern states (eg, Arunachal Pradesh and Himachal Pradesh). Approximately 50% of the geographic area in the country where over 70% population reside had incidence over 100 cases per 100 000 person-years; we found that less than 10% of the geographic area of the country with approximately 25% of the country population had incidence over 500 cases per 100 000 person-years.

**Table 2.** Predicted Incidence of Typhoid Fever at a State Level in India

State	Incidence (95% UI)	%Urban Population
Andaman and Nicobar	232 (196–324)	31.5
Andhra Pradesh	390 (321–534)	38.4
Arunachal Pradesh	204 (175–286)	19.2
Assam	166 (144–235)	15.2
Bihar	162 (140–230)	13.8
Chandigarh	905 (719–1228)	90.7
Chhattisgarh	305 (253–421)	29
Dadra and Nagar Haveli	446 (369–607)	45.5
Daman and Diu	564 (455–768)	72.3
Delhi	1245 (963–1702)	97.2
Goa	400 (339–540)	56.6
Gujarat	457 (374–624)	44
Haryana	393 (326–536)	39.7
Himachal Pradesh	149 (130–213)	13.2
Jammu and Kashmir	249 (210–346)	24.1
Jharkhand	298 (248–411)	28.1
Karnataka	441 (362–602)	43.3
Kerala	429 (356–582)	44.4
Madhya Pradesh	305 (255–419)	30.7
Maharashtra	515 (418–703)	48.4
Manipur	342 (283–470)	32.8
Meghalaya	254 (211–354)	22.3
Mizoram	444 (359–609)	45.4
Nagaland	264 (224–365)	26.7
Orissa	224 (190–313)	20.8
Puducherry	659 (521–904)	55.5
Punjab	427 (353–580)	43.9
Rajasthan	307 (256–424)	30
Sikkim	199 (174–276)	21.8
Tamil Nadu	494 (407–669)	50.2
Tripura	285 (237–394)	26.4
Uttar Pradesh	282 (235–390)	27
Uttaranchal	360 (302–490)	37.4
West Bengal	395 (323–543)	36.2
Country average	360 (297–494)	34.3

Abbreviations: UI, uncertainty interval.

All incidence estimates are presented as cases per 100 000 persons

### Sensitivity Analysis

We performed multiple sensitivity analyses to determine robustness of the model prediction. We tested the effect of removing 1 study site on the overall typhoid incidence prediction, which had modest effect on the national typhoid incidence estimate. The result of sensitivity analysis was presented in Supplementary Table S4 in the Appendix. In this analysis, we found that removal of Vellore had the largest effect on the estimate. We also tested alternative variables in the prediction model for typhoid incidence. Using a 2-variable model (ie, urban prevalence and improved toilet access), we estimated a national incidence of 364 cases per 100 000 person-years (95% CI, 287–530). A map of state variation is available in Supplementary Figure S2 in the Appendix.

## Discussion

In this study, we used a geospatial statistical model to estimate the incidence of typhoid fever in India. We computed a national incidence of approximately 360 cases per 100 000 person-years with higher burden in urban centers, corresponding to 4.5 million cases and 8900 deaths annually in India. This study utilized statistical modeling of incidence data on typhoid fever from 4 cohort and 6 hybrid surveillance studies, while incorporating data from a national health survey to use as predictors of typhoid incidence. The key study limitation was a modest sample size to calibrate our model, which mainly relied on urbanicity alone to predict typhoid fever incidence. Our national estimate on incidence of typhoid fever in India is generally consistent with previous studies and supports that there is a large burden of typhoid fever in India that would benefit from national vaccination.

Our findings suggest substantial variation of typhoid incidence across the country. We found higher incidence in urban centers in the north and southwestern states and lower incidence in northern rural regions driven largely by the single model variable of urbanicity. This urban-rural disparity highlights that the burden of typhoid fever in India is predominately in larger urban centers that may be related to living conditions such as density, sanitation, and other environmental factors, although there was still typhoid fever found in rural areas. Prior epidemiologic work has demonstrated the risk of typhoid fever in urban centers [9, 19]. This suggests a higher risk in these areas, which may support prioritization of vaccination in these settings [37]. We also found a relationship between typhoid incidence and growth metrics, vaccination, and improved toilet/water; some of these variables may have a causal relationship with typhoid fever infection, but they also may only be correlated with risk factors for typhoid fever, and this analysis is limited by a small sample size.

WHO now recommends introducing typhoid conjugate vaccines in areas with high typhoid incidence [38]. Prior cost-effectiveness models have found that typhoid conjugate vaccines would be cost-effective in routine immunization programs for countries with high typhoid incidence [39, 40, 41]. The Government of India is preparing to make a decision about whether and how to introduce typhoid conjugate vaccines. Data on the burden of typhoid fever across the country are important to support these decisions. Despite substantial spatial variation, we estimated that the incidence of typhoid in all states were likely above 100 per 100 000 person-years, which has been defined as “high burden” for typhoid [3]. These findings suggest that a nationwide vaccine introduction, rather than a geographically targeted one, may be required for control of typhoid and mitigation of its health impacts in India.

The study findings should be interpreted within the limitations of the data and analysis. The study had a key limitation in sample size; we used 10 sites to calibrate the model to predict typhoid fever incidence across India, which limited the accuracy and validation of the model prediction. Measurement of typhoid fever incidence at a given location requires a resource-intensive methodology (eg, multiyear cohort study or hybrid surveillance), which limits the number of locations where typhoid incidence can be reliably estimated. We adjusted our case data for an estimated 60% sensitivity of blood cultures for diagnosis of typhoid fever. However, this adjustment could be limited because blood culture sensitivity varies across locations, and it could further result in underestimation or overestimation of typhoid incidence in certain areas. To address the concern of the influence of a single site on the overall national typhoid estimate given a small sample size, we performed sensitivity analyses and found that 1 site did not disproportionately affect the estimate. We relied upon secondary data from the DHS to predict typhoid incidence; these data required some data processing, interpolation, and aggregation to a grid level (5 × 5 km), which could introduce imprecision and bias into the estimate. An independent assessment of DHS data was done through comparison to the state urban population data from the Indian Ministry of Home Affairs [42]. Supplementary Table S5 in the Appendix shows our computed urban population and data from the national India Census, which were overall comparable. We constrained the number of predictors in the model to limit the risk of overfitting given limited data, both of which limited the model’s predictive accuracy (Table 3). The model identified a negative association between typhoid incidence and being stunted or underweight (Table 1). Our urban sites (eg, Chandigarh, Delhi, Kolkata, and Vellore) had substantially higher typhoid incidence than rural sites, yet stunting and underweight are much less common in urban locations, which likely explains this negative association. Due to the small sample size for the model calibration, we were unable to perform a meaningful validation of the model prediction. In addition, the 4 study sites in Tier 1 included all children, and 3 of these sites were in urban settings. Because typhoid fever has greater risk for children, this site selection could bias our estimate towards a higher incidence given the urban settings and pediatric population included in this study. Estimation bias could have also resulted from the differences in the time frame between sample collection for SEFI and DHS. Finally, due to a limited sample size, we did not include age-specific incidence estimates, although there is likely a strong age correlate of risk.

**Table 3.** Comparison of observed and predicted incidence of typhoid fever in SEFI study sites

Site	Original		Predicted	
	Incidence	95 %UI	Incidence	95 %UI
Anantapur	266	(176–412)	400	(334–543)
Chandigarh	981	(717–1416)	941	(744–1280)
Delhi	1095	(913–1302)	1313	(1010–1799)
East Champaran	72	(50–113)	80	(71–124)
Karimganj	79	(59–133)	96	(86–144)
Kolkata	1187	(998–1400)	1313	(1010–1799)
Kullu	274	(179–443)	274	(239–371)
Nandurbar	154	(98–280)	137	(120–198)
Vadu	61	(24–125)	192	(174–263)
Vellore	1977	(1740–2236)	1185	(926–1613)

The original typhoid incidence was provided by SEFI. The predicted incidence for each site was based on the model prediction. All incidence estimates are presented as cases per 100 000 person-years.

Abbreviations: SEFI, Surveillance for Enteric Fever in India; UI, uncertainty interval

## Conclusions

There is a substantial disease burden of typhoid fever across India, with higher typhoid incidence in urban centers. This study supports immunization with the Vi conjugate typhoid vaccine to address the disease burden from typhoid fever in India.

## Supplementary Data

Supplementary materials are available at *The Journal of Infectious Diseases* online. Consisting of data provided by the authors to benefit the reader, the posted materials are not copyedited and are the sole responsibility of the authors, so questions or comments should be addressed to the corresponding author.

jiab187_suppl_Supplementary_Appendix

## Notes

***Acknowledgments.*** We thank the study participants and research team who assisted with the Surveillance for Enteric Fever in India (SEFI) studies.

***Author contributions.*** Y. C. and N. C. L. had full access to all of the data in the study and take responsibility for the integrity of the data and the accuracy of the data analysis. G. K., J. J., J. R. A., and N. C. L. contributed to study conception. All authors contributed to study design, data analysis, and data interpretation. Y. C. and N. C. L. wrote the first draft. All authors contributed to intellectual material and approved the final draft.

***Disclaimer.*** The funding organizations had no role in the design and conduct of the study; collection, management, analysis, and interpretation of the data; and preparation, review, or approval of the manuscript; or the decision to submit the manuscript for publication.

***Supplement sponsorship.*** This supplement is sponsored by the Christian Medical College Vellore Association.

***Financial support.*** This study was funded by the Bill & Melinda Gates Foundation (OPP1159351). N. C. L. is supported by the University of California, San Francisco (Department of Medicine).

***Potential conflicts of interest.*** N. C. L. reports personal fees from the World Health Organization, unrelated to the present work. All authors have submitted the ICMJE Form for Disclosure of Potential Conflicts of Interest. Conflicts that the editors consider relevant to the content of the manuscript have been disclosed.
