# Supplementary material for: Geographic Pattern of Typhoid Fever in India: A Model-Based Estimate of Cohort and Surveillance Data
Source: J Infect Dis. 2021 Nov 23;224(Suppl 5):S475–83. doi: 10.1093/infdis/jiab187 (PMC8892532; doi:10.1093/infdis/jiab187)
Supplement: jiab187_suppl_Supplementary_Appendix [file jiab187_suppl_supplementary_appendix.docx]

**Supplemental Materials**

Cao Y, Karthikeyan AS, Ramanujam K, Raju R, Krishna S, Dilesh K, Ryckman T, Mohan VR, Kang G, John J, Andrews JR and Lo NC. Geographic pattern of typhoid fever in India: a model-based estimate of cohort and surveillance data.

**Contents**

Section 1: Technical appendix.……………..…………….…….….………………..………Page 3

Section 2: Supplemental tables …………………..….…………………..…………….....…Page 4

Section 3: Supplemental figures …………………..…………….…………….....…….....…Page 9

- **Section 1: Technical appendix**

In this supplement, we provide additional methodological details on the statistical modeling of typhoid incidence.

In the cohort study, the number of typhoid cases was collected for each study site (4 sites in total). The incidence rate (per 100,000 population) was computed for each site as $\frac{N\_typhoid}{N\_child\_years}*$100,000, where $N\_typhoid$ refers to the number of typhoid cases and $N\_child\_years$ refers to the number of person-years of observation in the defined age group, with follow up censored for 15th birthday, withdrawal of consent/assent, febrile period, death, and completion of study.

In the hybrid study, the number of typhoid cases was collected in the 6 study sites. The incidence rate (per 100,000 population) was estimated as $\frac{N\_typhoid}{N\_person\_years}*\frac{1}{care\_seek}*100,000$, where $N\_typhoid$ refers to the number of typhoid cases, $N\_person\_years$ refers to the number of person-years of observation in each study site. $care\_seek$ denotes the care seeking probability in each study site.

- **Section 2: Supplemental tables**

Table S1. Data description for variables from Demographic and Health Survey

| **Covariate description** | **Category** | **Data source** | **Data description** | **Data type for child** | **Data type for household** | **Data type for cluster** |
| --- | --- | --- | --- | --- | --- | --- |
| Urban prevalence | SES | DHS | Urban percent |  |  | Binary |
| Wealth | SES | DHS | Wealth index |  | Categorical |  |
| Maternal education level | SES | DHS | Educational achievement recodes the education of the respondent |  | Categorical |  |
| Household size | Household risk factor | DHS | Total number of household members is the number of usual residents plus the number of visitors who slept in the house the previous night that were listed in the household schedule |  | Continuous |  |
| Improved water access | Household risk factor | DHS | Main source of drinking water for members of the household. |  | Binary |  |
| Improved toilet access | Household risk factor | DHS | Type of toilet facility in the household. |  | Binary |  |
| 3rd dose DPT vaccine | Healthcare access | DHS | DPT3 (diphtheria, pertussis and tetanus) vaccination | Binary |  |  |
| Stunting | Healthcare access, SES | DHS | Std.dev. for height/age | Continuous –reclassified to binary |  |  |
| Underweight | Healthcare access, SES | DHS | Std.dev. for weight/age | Continuous –reclassified to binary |  |  |

Table S2. Hierarchical recoding and computation for DHS variables

| **Covariate description** | **Data recode for child** | **Data recode for household** | **Computation at cluster level** | **Data type at cluster level** |
| --- | --- | --- | --- | --- |
| Urban prevalence |  |  | Rural: 0  Urban: 1 | Rural: 0  Urban: 1 |
| Wealth |  | Poorest: 1  Poorer: 2  Middle: 3  Richer: 4  Richest: 5 | Mean for all households | Mean wealth index (1-5) |
| Maternal education level |  | No education: 1  Incomplete primary/complete primary: 2  Incomplete secondary/complete secondary/higher: 3 | Mean for all households | Mean education index (1-3) |
| Household size |  |  | Mean for all households | Mean number |
| Improved water access |  | Not improved: 0  Improved: 1 | Mean for all households | Improved water prevalence (%) |
| Improved toilet access |  | Not improved: 0  Improved:1 | Mean for all households | Mean improved toilet prevalence (%) |
| 3rd dose DPT vaccine | No: 0  Vaccination on card: 1  Reported by mother: 1  Vaccination marked on card: 1  Don’t know/NA: Not counted |  | Mean for all children | Vaccine coverage (%) |
| Stunting | >=-200: 0  < - 200: 1 |  | Mean for all children | Stunting prevalence (%) |
| Underweight | >=-200: 0  < - 200: 1 |  | Mean for all children | Underweight prevalence (%) |

Table S3 Classification for improved water and improved toilet access

| Water access | | Toilet access | |
| --- | --- | --- | --- |
| **Improved** | **Unimproved** | **Improve** | **Unimproved** |
| piped into dwelling | unprotected well | flush to septic tank | no facility/bush/field |
| piped to yard/plot | river/dam/lake/ponds/stream/canal/irrigation channel | flush, don't know where | not a de jure resident |
| public tap/standpipe | unprotected spring | flush to piped sewer system | pit latrine without slab/open pit |
| tube well or borehole | other | flush to pit latrine | dry toilet |
| protected well | not resident | flush to somewhere else | other |
| Protected spring | tanker truck | composting toilet |  |
| bottled water | cart with small tank | ventilated improved pit latrine |  |
| rainwater |  | pit latrine with slab |  |
| community |  |  |  |

Table S4 Sensitivity test of national incidence estimate by removing one site

| Site removed | National incidence | 95%UI |
| --- | --- | --- |
| Anantapur | 354 | 288 – 493 |
| Chandigarh | 383 | 302 – 535 |
| Delhi | 374 | 300 – 533 |
| East Champaran | 361 | 294 - 506 |
| Karimganj | 360 | 290 – 504 |
| Kolkata | 368 | 290 – 531 |
| Kullu | 350 | 290 – 478 |
| Nandurbar | 361 | 292 – 500 |
| Vadu | 364 | 301 – 500 |
| Vellore | 330 | 375 - 456 |

*The national incidence returned from the model was 360 (379, 494). The above result shows when removing Vellore, the national incidence differs the most.

Table S5 Percentage of urban population – independent assessment on DHS data

|  | % of urban population | |
| --- | --- | --- |
| State | DHS data based | Indian Census based |
| Andaman and Nicobar | 31.5 | 35.7 |
| Andhra Pradesh | 38.4 | 33.5 |
| Arunachal Pradesh | 19.2 | 22.7 |
| Assam | 15.2 | 14.1 |
| Bihar | 13.8 | 11.3 |
| Chandigarh | 90.7 | 97.3 |
| Chhattisgarh | 29 | 23.2 |
| Dadra and Nagar Haveli | 45.5 | 46.6 |
| Daman and Diu | 72.3 | 75.2 |
| Delhi | 97.2 | 97.5 |
| Goa | 56.6 | 62.2 |
| Gujarat | 44 | 42.6 |
| Haryana | 39.7 | 34.8 |
| Himachal Pradesh | 13.2 | 10 |
| Jammu and Kashmir | 24.1 | 27.2 |
| Jharkhand | 28.1 | 24.1 |
| Karnataka | 43.3 | 38.6 |
| Kerala | 44.4 | 47.7 |
| Madhya Pradesh | 30.7 | 27.6 |
| Maharashtra | 48.4 | 45.2 |
| Manipur | 32.8 | 30.2 |
| Meghalaya | 22.3 | 20.1 |
| Mizoram | 45.4 | 51.5 |
| Nagaland | 26.7 | 28.9 |
| Orissa | 20.8 | 16.7 |
| Puducherry | 55.5 | 68.3 |
| Punjab | 43.9 | 37.5 |
| Rajasthan | 30 | 24.9 |
| Sikkim | 21.8 | 24.9 |
| Tamil Nadu | 50.2 | 48.5 |
| Tripura | 26.4 | 26.2 |
| Uttar Pradesh | 27 | 22.3 |
| Uttaranchal | 37.4 | 30.6 |
| West Bengal | 36.2 | 31.9 |

*Indian Census data were retrieved from Indian Ministry of Home Affairs

**Section 3: Supplemental figures**


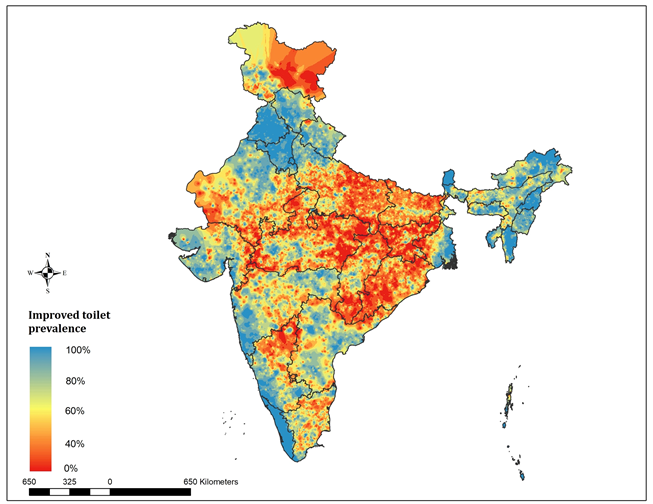

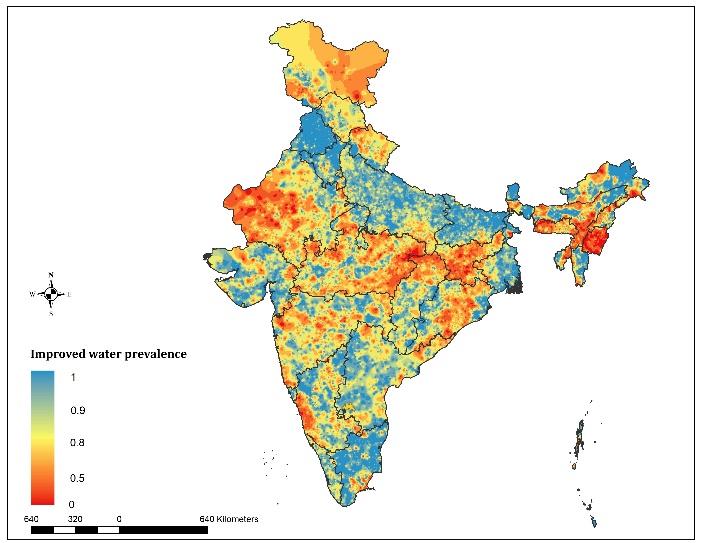


(a) RMSE = 0.23 (b) RMSE = 0.17


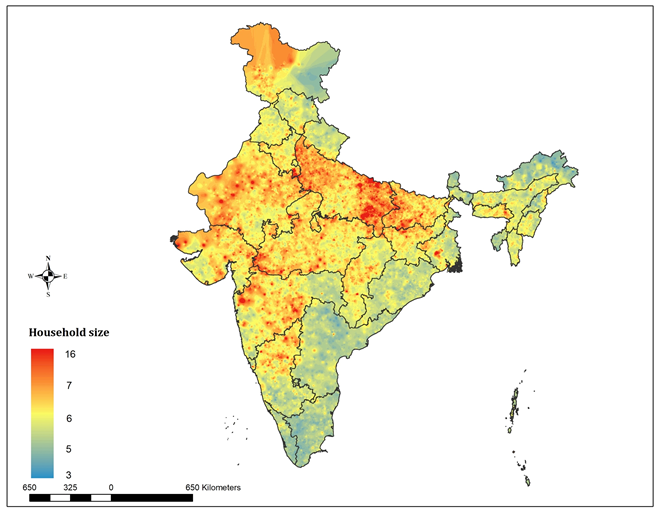

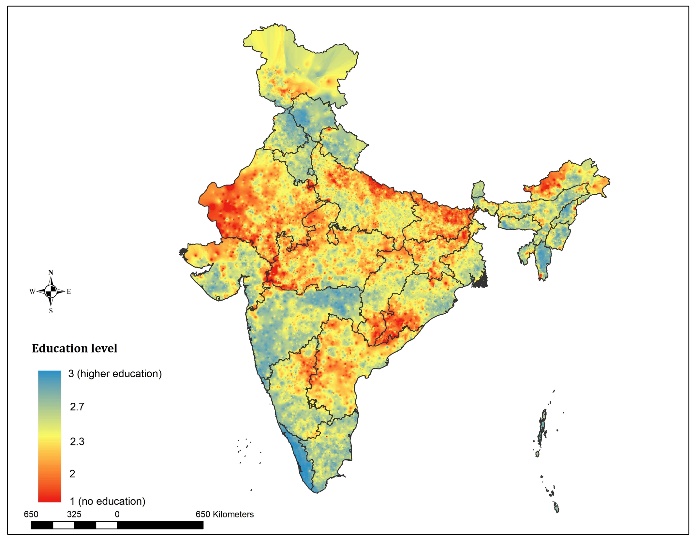


(c) RMSE = 1.02 (d) RMSE = 0.32


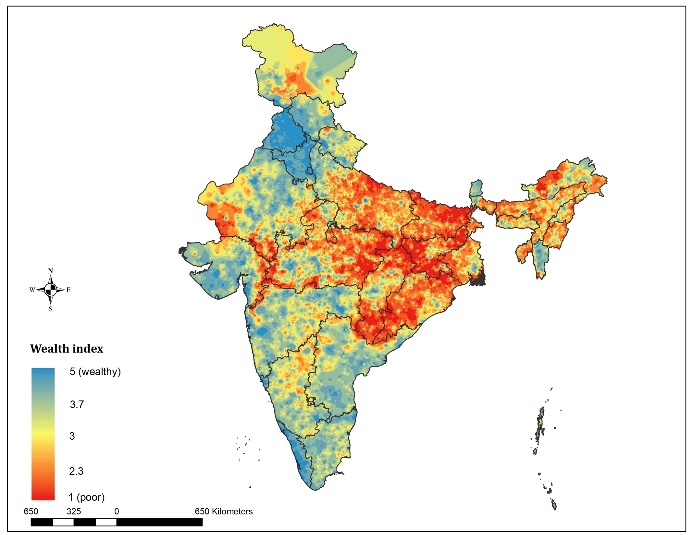

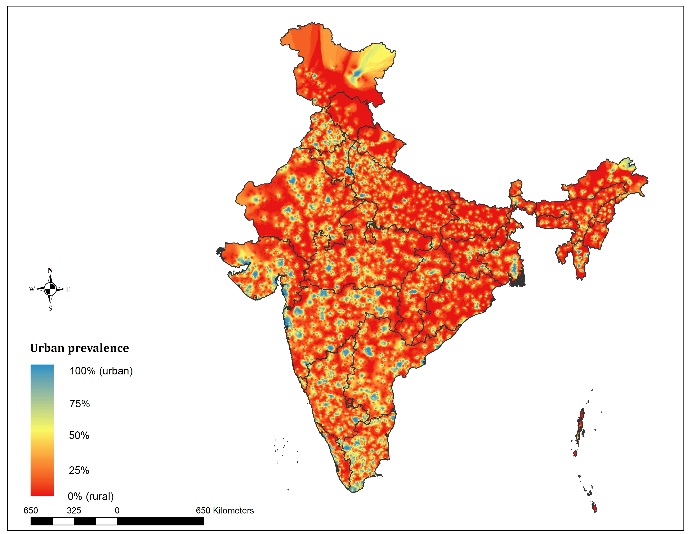


(e) RMSE = 0.66 (f) RMSE = 0.34


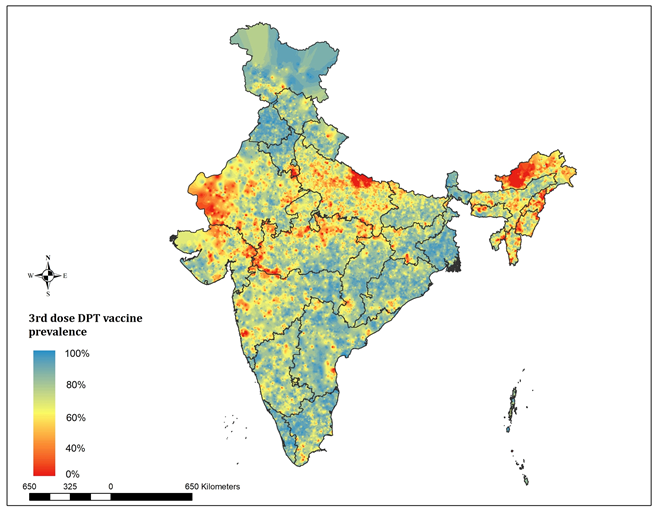

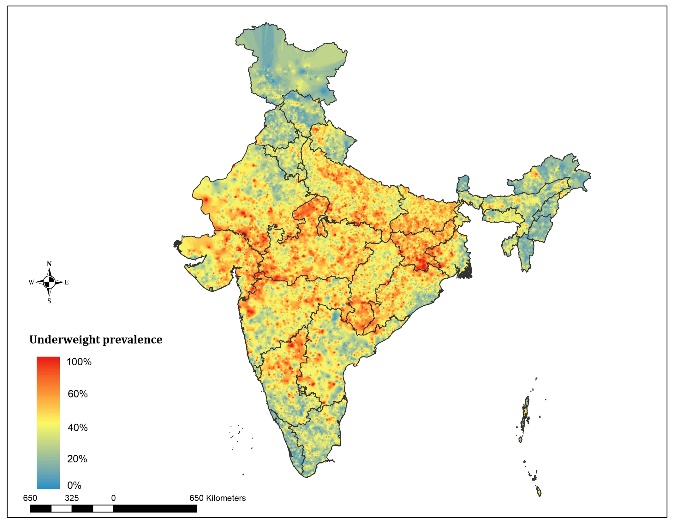


(g) RMSE = 0.24 (h) RMSE = 0.23


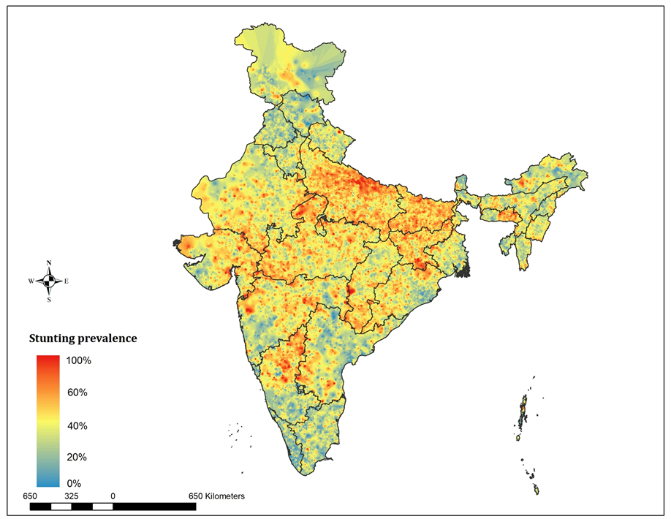


(i) RMSE = 0.24

**Figure S1: Spatial interpolation of DHS variables using inverse distance weighting**. Estimate available for (a) improved toilet prevalence; (b) Improved water prevalence; (c) household size; (d) education level; (e) wealth index; (f) urban prevalence; (g) 3^rd^ dose DPT vaccine prevalence; (h) underweight prevalence; (i) stunting prevalence


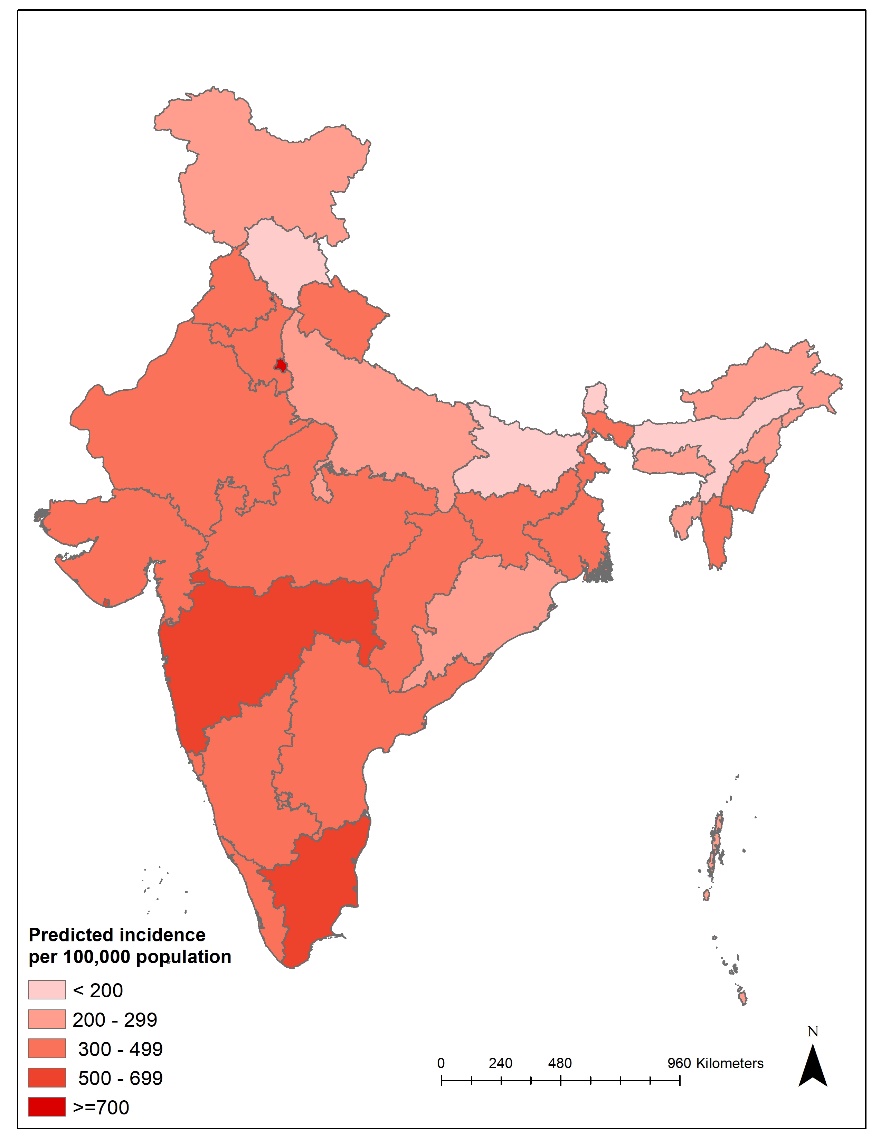


**Figure S2: Predicted incidence of typhoid fever in India at state level**. We used two predictors in this model: urban prevalence and prevalence for improved toilet access
